# Identifying the Spatial Imbalance in the Supply and Demand of Cultural Ecosystem Services

**DOI:** 10.3390/ijerph19116661

**Published:** 2022-05-30

**Authors:** Qinqin Shi, Hai Chen, Di Liu, Tianwei Geng, Hang Zhang

**Affiliations:** 1 Cooperative Innovation Center for Transition of Resource-Based Economics, Shanxi University of Finance & Economics, Taiyuan 030006, China; shiqinqin_1314@126.com; 2 Research Institute of Resource-Based Economics, Shanxi University of Finance & Economics, Taiyuan 030006, China; 3 College of Urban and Environmental Sciences, Northwest University, Xi’an 710127, China; gengtianwei1002@126.com; 4 School of Culture and Tourism, Ningxia University, Zhongwei 755000, China; lcx@stumail.nwu.edu.cn; 5 Institute of Land and Urban-Rural Development, Zhejiang University of Finance and Economics, Hangzhou 310018, China; zhrwdl2000@126.com

**Keywords:** cultural ecosystem services, supply, demand, spatial imbalance, tradeoffs/synergies

## Abstract

Cultural ecosystem services (CESs) are an important part of ecosystem services (ESs). Correctly understanding the supply and demand relationship of CES is the premise of ES sustainable management and helps to improve human well-being. However, the evaluation and mapping of CES supply and demand represents a significant gap in ES research. Using the Shigou Township of Mizhi County in China as an example, in this study, we evaluated CES supply and demand at the village scale. We first considered three aspects of supply potential, accessibility and quality to construct an indicator system of six types of CES supply, including aesthetic (Aest), sense of place (SP), social relations (SR), cultural heritage (Cult), education (Edu) and recreation (Recr) and obtained demand data through a questionnaire. Then, we identified the imbalance in the supply and demand of CES by Z-score standardization based on the quantification of the CES supply and demand. Secondly, bivariate spatial autocorrelation analysis was used to identify tradeoffs/synergies on the CES supply side, and chi-square tests were used to identify CES demand differences between stakeholder groups. The results indicated that the supply–demand patterns of CES presented evident spatial differences. The low-supply–high-demand patterns of Aest, SR and Recr accounted for the largest proportions, with values of 33.33%, 33.33% and 30.95%, respectively. The low-supply–low-demand patterns of SP and Cult accounted for the largest proportions, with values of 30.95% and 38.10%, respectively. The low-supply–low-demand pattern of Edu accounted for the smallest proportion (21.43%) and was mainly located in the south of Shigou Township. The southwest, northeast and central areas of Shigou Township were the key regions of tradeoffs/synergies of CES supply. There were significant differences in CES demand for SR, Cult and Edu among stakeholder groups. The results could contribute to optimizing regional ecosystem management and provide effective information for improving the imbalance between the supply and demand of CES.

## 1. Introduction

Ecosystem services (ESs) are the foundation of human survival and development [1]. The sustainable management of ESs should not only ensure the capacity of ecosystems to maintain their long-term supply of ESs but also consider growing social demand [2]. In recent years, the evaluation of the supply and demand of ESs has become a global research focus [3,4]. As one of the four pillars of ES, cultural ecosystem services (CESs) are regarded as the non-material benefits generated by the interactions between social and ecological systems [5,6], which are directly experienced and intuitively appreciated by people [7]. These services have established a solid connection between human beings and the natural environment and have become a strong motivator for people to participate in the protection of natural capital [8,9]. As an important bridge connecting ecosystems and social systems, CES involves the interaction between ecosystem and social factors. The evaluation of CES must consider both the ability of the ecosystem to provide these services and human demand for these services [10]. A comprehensive evaluation of cultural services from the perspective of supply and demand is helpful to provide theoretical support for ecosystem management, rational allocation of natural resources and harmonious development between man and nature [11].

In recent years, the study of CES has attracted increasing attention from scholars, and the evaluation methods of CES have also been enriched [12]. The evaluation methods of CES include the ecosystem services value method [13], the ES matrix method based on land-use types [14], the social preference method [15], the participatory GIS mapping method [16,17], the social media-based method [18], the biophysical indicator evaluation method [19], etc. Among them, the ecosystem services value method is based on expert knowledge and land-use data for monetary valuation of ecosystem services. It is intuitive and easy to use and is especially suitable for the assessment of ecosystem services at regional and global scales. This method has been widely used [20,21], but includes limited types of CES, such as leisure, culture and landscape aesthetics [13,22]. The ES matrix method connects land-use types with CES and provides a useful tool for the quantification and spatial display of various types of CES. It has been applied to many case sites [23,24,25]. However, because the method is based on expert experience, knowledge and attitudes, the research results are inevitably subjective to some extent, and the impact of factors other than land use on CES should also be considered in future research [26]. The social preference method adopts direct inquiry to judge the social importance of CES by analyzing people’s social motivation, cognition and related value of CES [15], including focus group discussions, narrative and questionnaire methods, etc. [12]. This method helps to reveal the value hidden by the monetary method and can also solve the problem of missing data in the study area [15] and comprehensively analyze various types of CES, such as aesthetics, sense of place, education, spirituality, etc. [12,27]. The participatory GIS mapping method allows participants to directly point out the location and content of CES on a map and conduct digital analysis in combination with geographic information systems (GIS) so as to finally realize the spatial mapping of CES [17]. This method usually adopts the social values for ecosystem services (SolVES) or maximum entropy (Maxent) model to realize the value transfer of CES [16]. The method can improve the legitimacy and acceptance of the decision-making and planning process by actively engaging the public, but it is usually high-cost and time-consuming and can only be investigated in specific and limited areas [28]. The social-media-based method is used to obtain photo data with geographical coordinates on network platforms such as Panoramio, Flickr, Google Earth or GAODE map and realize the spatialization of CES through kernel density, hotspot analysis and the Maxent model [18,29,30]. This method can help identify the spatial location where people enjoy CES and realize the spatial mapping of CES on a regional scale. The disadvantage is that there may be problems with the representativeness of the samples, as the use of social media is affected by the regional internet usage rate; GPS camera and mobile phones; and individuals’ age, education level and ability to use social networks [31]. The biophysical indicator evaluation method provides objective quantitative data and clear spatial details based on the process and function of the ecosystem, which can provide intuitive and effective information for ecosystem management and decision making. It has been increasingly used in the evaluation of ES supply [30,32]. However, there are too few indicators for CES supply evaluation [10,33].

With the diversity and improvement of CES evaluation methods, some scholars began to pay attention to the integrated assessment of CES supply and demand [31,34]. CES supply is influenced by natural geographical factors in space, and CES demand is determined by the social and economic activities of individuals or groups. Therefore, CES supply and demand have strong spatial heterogeneity and imbalanced characteristics [35,36]. How to improve the imbalance in the supply and demand of CES is a considerable challenge associated with achieving a sustainable CES supply and meeting human demand in a given region [37]. In the literature, the most common assessment of supply and demand focuses on aesthetic and recreation services. For example, Cui et al. [38] evaluated the supply and demand of landscape aesthetics in Hulunbuir, China, by using a visual quality index and the number of tourists per unit area to represent aesthetic supply and demand, respectively. Baró et al. [39] mapped the supply and demand of outdoor recreation in the Barcelona metropolitan region and mapped the outdoor recreation supply based on three components—degree of naturalness, nature protection and presence of water—and mapped the outdoor recreation demand by modeling the number of visitors that reached a given recreational area within a given distance threshold. However, in general, an easy-to-implement method at the operational level for the integrated assessment of various types of CES supply and demand is lacking.

The UK National Ecosystem Assessment Follow-on (UK NEAFO) includes an indicator system for CES supply based on the percentage of land cover of a range of landscapes, obtaining CES demand data through an ongoing, face-to-face, in-home omnibus survey [6]. The combination of biophysical indicators and public participation methods provides a reference for the quantification of CES supply and demand. In addition, scholars have noted that the tradeoffs between different ESs and the demand difference among stakeholders are among the important reasons causing the imbalance between the ES supply and demand [2,40]. However, on the basis of quantifying the supply and demand of various types of CES and identifying the imbalance between the supply and demand of CES, there is still a gap to further explore the tradeoffs between different types of CES on the supply side and the demand difference among stakeholders [41].

Therefore, in this study, we evaluated CES supply and demand by coupling biophysical indicators and questionnaire methods to optimize regional ecosystem management and provide effective information for improving the imbalance between the supply and demand of CES. The specific objectives of this study are as follows:

(1) To evaluate various types of CES supply and demand by coupling biophysical indicators and questionnaire methods;
(2) To identify the imbalance in the supply and demand of CES in order to reveal the spatial tradeoffs/synergies between the six types of CES on the supply side, as well as the demand differences between stakeholder groups on the demand side;
(3) To propose strategies for improving the imbalance between the supply and demand of CES in the study area.

First, a supply indicator system of six types of CES, including aesthetic (Aest), sense of place (SP), social relations (SR), cultural heritage (Cult), education (Edu) and recreation (Recr), was constructed. The demand of CES was calculated with questionnaire data. Secondly, the imbalance in the supply and demand of CES was identified by the Z-score standardization method, the spatial tradeoffs/synergies between the six types of CES on the supply side were revealed by bivariate local Moran’s I and the demand differences between stakeholder groups on the demand side were revealed by the chi-square tests. Finally, we provide specific suggestions for improving the imbalance between the supply and demand of CES in the study area.

## 2. Materials and Methods

### 2.1. Study Area: Shigou Township

**Figure 1:**
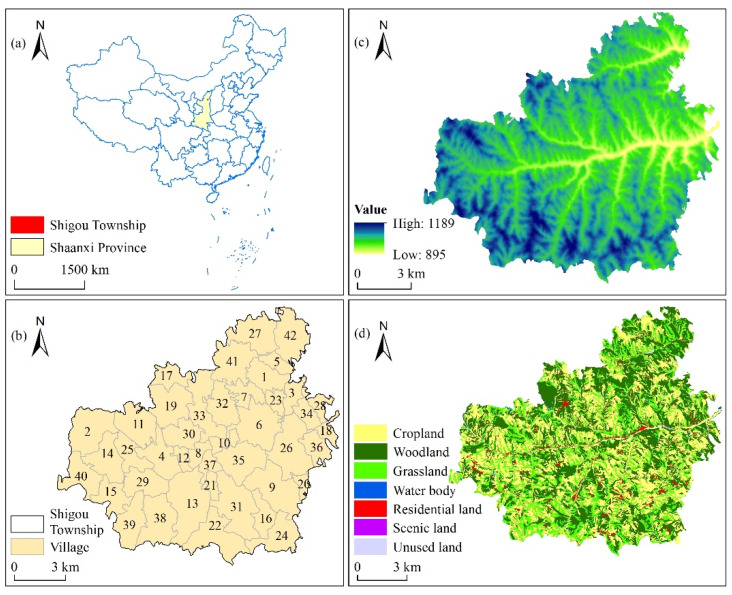
Map of the study area: (**a**) location of Shaanxi Province, China; (**b**) 42 villages of Shigou Township; (**c**) the digital elevation model (DEM) of Shigou Township; (**d**) spatial distribution of land use in Shigou Township.

Shigou Township is located 12.5 km west of Mizhi County, Shaanxi Province (Figure 1). It is a demonstration township for watershed governance and ecological environment protection in Mizhi County, with an administrative area of 151.76 km^2^ [42]. Shigou Township has 42 administrative villages, with 6433 households and a population of 17,243 [43]. The township has a total arable land area of 77.87 km^2^ [44], with abundant forest and grassland resources; the coverage rate of forest and grassland was 45.7% as of 2017 [43]. Shigou Township is a typical rural area developed by a combination of agriculture and tourism. The development model of agriculture is “mountain apples + small grain crops (millet and beans) + breeding industry (sheep)”. As a famous tourist town, Shigou Township is rich in tourism resources, including the“Liujiagua canyon scenic spot”, “Hill Gorge on the Loess Plateau”, the “Diaochan Cave” and the red revolutionary education base “Dushi Memorial Hall”. Since the implementation of the “Grain for Green” policy in 1999, the land use in Shigou Township has undergone considerable changes; the area of cultivated land has been reduced [45], and the labor force has experienced surplus and annual emigration of nearly 6000 people from the township [44]. Subsequently, the traditional cultural landscapes of the township have subtly changed, and many valuable cultural and ecological elements have been gradually lost, resulting in increasing conflict between the supply and demand of CES. Therefore, the quantification and spatial mapping of CES in Shigou Township are of considerable significance for ecosystem management in this region.

### 2.2. Data

(1) The land-use data were acquired from the Chinese Academy of Sciences Resource Environmental Data Center. The data were acquired from high-resolution images of GF-1 captured in 2018 by visual interpretation and digitalization. Visual interpretation refers to the typical ground features and data from the second national land survey conducted by the Ministry of Land and Resources. The average kappa coefficient reached 0.86, and a 10 m × 10 m grid was resampled. Referring to the National Standard Land-Use Classification of China (GB/T21010-2017) and the land-use conditions in the study area, the land-use classes were divided into seven types: cropland, woodland, grassland, water body, residential land, scenic land and unused land.
(2) Normalized difference vegetation index (NDVI) data were derived from Landsat 8 remote-sensing images (15 m panchromatic and 30 m multispectral) from August 2018 of the Geospatial Data Cloud (http://www.gscloud.cn, accessed on 15 August 2018). ENVI 5.1 software (Harris Geospatial Solutions, Broomfield, CO, USA) was used to process radiation calibration and atmospheric correction on the multispectral bands and fused with the panchromatic bands to form a 10 m resolution image.
(3) Other CES supply data were acquired through interviews with government staff in each village, including data on the village population, the number of households, area of cave dwellings, area of temples and cultural activity centers, area of “three types of land” (terraced, dam and irrigated land) [46] and area of “Grain for Green”. See the Supplementary Materials (Questionnaire S2) for details.

Other CES demand data were obtained from questionnaires. The questionnaire survey was supported by the research of the National Natural Science Foundation, which simulated urban expansion in oases based on supply, demand and flows of ecosystem services. The research team went to Shigou Township in Mizhi County in June 2018 and randomly selected five villages for pre-survey. The pre-survey found that local residents have a limited understanding of CES, ultimately revealing six types of popular CESs in the study area, including Aest, SP, SR, Cult, Edu and Recr. We revised and improved the questionnaire according to the results of the pre-survey. From July to August 2018, our research team conducted a formal questionnaire survey of all 42 villages in Shigou Township and obtained 386 questionnaires from face-to-face interviews; 381 questionnaires were valid, with an effective rate of 98.7%. The content of the questionnaire was divided into two parts. The first part was a brief description of the respondents, including age, gender, education level and occupation. The second part comprised respondents’ perceptions of the importance of multiple CESs, which were measured on a 5-point Likert scale ((1): very low importance; (2): low importance; (3): medium importance; (4): high importance; (5): very high importance). See the Supplementary Materials (Questionnaire S1) for details.

### 2.3. Methods

#### 2.3.1. Quantifying CES Supply

(1) Construction of the CES supply indicator system

Although the classification of CES is clear, it is difficult to calculate each type of CES separately. It is difficult to assign an element or function of the ecosystem to a certain type of CES [47]. For example, forests can provide aesthetic services, as well as recreational services. To avoid double calculation, different CESs should be assigned to different landscapes before CES evaluation. For example, when forest is allocated as a measure of aesthetics, it cannot be included in recreational calculations [48]. Based on this principle, referring to the indicator system of the UK NEAFO and considering the three aspects of supply, accessibility and quality, the land-use coverage, the distance from residential locations to each landscape and the landscape index were used as proxy data to measure the CES supply [6,49]. In this study, we took administrative villages as the research scale and considered data availability, as well as the main land-use types in Shigou Township. A CES supply indicator system was established for Shigou Township from the perspective of practical quantification and spatialization (Table 1).

Concretely, (1) aesthetics are the degree of pleasure derived from natural background conditions according to human visual perception, and naturalness and landscape heterogeneity are important factors affecting aesthetics [50]. Naturalness is represented by the NDVI value, which is commonly used for calculating vegetation coverage [51], and landscape heterogeneity is represented by Shannon’s diversity index (SHDI) [52]. (2) Sense of place refers to the inherent characteristics of the place and people’s attachment to the place, including the objective characteristics of the place and people’s subjective cognition [53]. Supply refers to the unique physical characteristics of the place. Shigou Township is a typical rural area dominated by agricultural civilization, and villagers interact closely with cropland. In addition, Shigou Township is located on the Loess Plateau, and cave dwellings are its unique residential form. Cropland and cave dwellings are space carriers that rely on the emotions of local residents. Therefore, the per capita cropland area and per household cave dwelling area are indicators representing the supply of sense of place. For example, Schröter et al. [54] argued that second homes (cabins) in Norway area social construct expressing emotional attachment to environmental surroundings. (3) Ecosystems influence the types of social relations that are established in specific cultures [1]. Many villages in China are formed on the basis of consanguinity and long-term settlement, and mutual assistance is a characteristic of China’s rural society [55]. Therefore, we used residential separation, distance to the nearest township and distance to the county of each village to indicate the impact of settlement layout and the convenience degree in relation to connecting with the outside world for internal and external social relations. (4) Cultural landscapes are important constituents of cultural heritage, carriers of cultural value and an important expression of community identity [56]. As the “hometown of culture” and “hometown of small drama”, Mizhi County emphasizes the inheritance of traditional cultural activities, as well as traditional cultural activities with local characteristics, such as “drama performance” and “temple fair”, which continue to this day [57]. Cultural activity centers and temples are the cultural landscapes of traditional cultural activities held in Shigou Township; thus, the areas of cultural activity centers and temples in each village are used as supply indicators of cultural heritage services. (5) Ecological protection has unique strategic significance for the Loess Plateau area, and the long-term construction of basic farmland with “three types of land” (terraced, dam and irrigated land) in Shigou Township has strengthened the capacity of water conservation, as well as the drought resistance of farmland [46]. Moreover, the “Grain for Green” project has significantly improved the vegetation and natural ecological environment of the township [58]. These ecological restoration projects provide educational services for the harmonious coexistence of humans and nature. Therefore, the percentage of “three types of land” area and “Grain for Green” area are taken as indicators of educational supply. (6) All ecosystems are potential providers of recreational services [39,59]. Landscape accessibility is a reflection of landscape usability and degree of human participation in the landscape [60]. In this study, the percentage of scenic areas and the shortest distance from residential locations to woodlands, grasslands and water bodies are taken as indicators of the recreational supply. In addition, during the investigation, it was found that cultural activity centers are public spaces for the daily entertainment of residents in Shigou Township. Therefore, the average distance from residential locations to the cultural activity center of each village was considered an indicator of recreational supply.

(2) Calculating the CES supply

**Figure 2:**
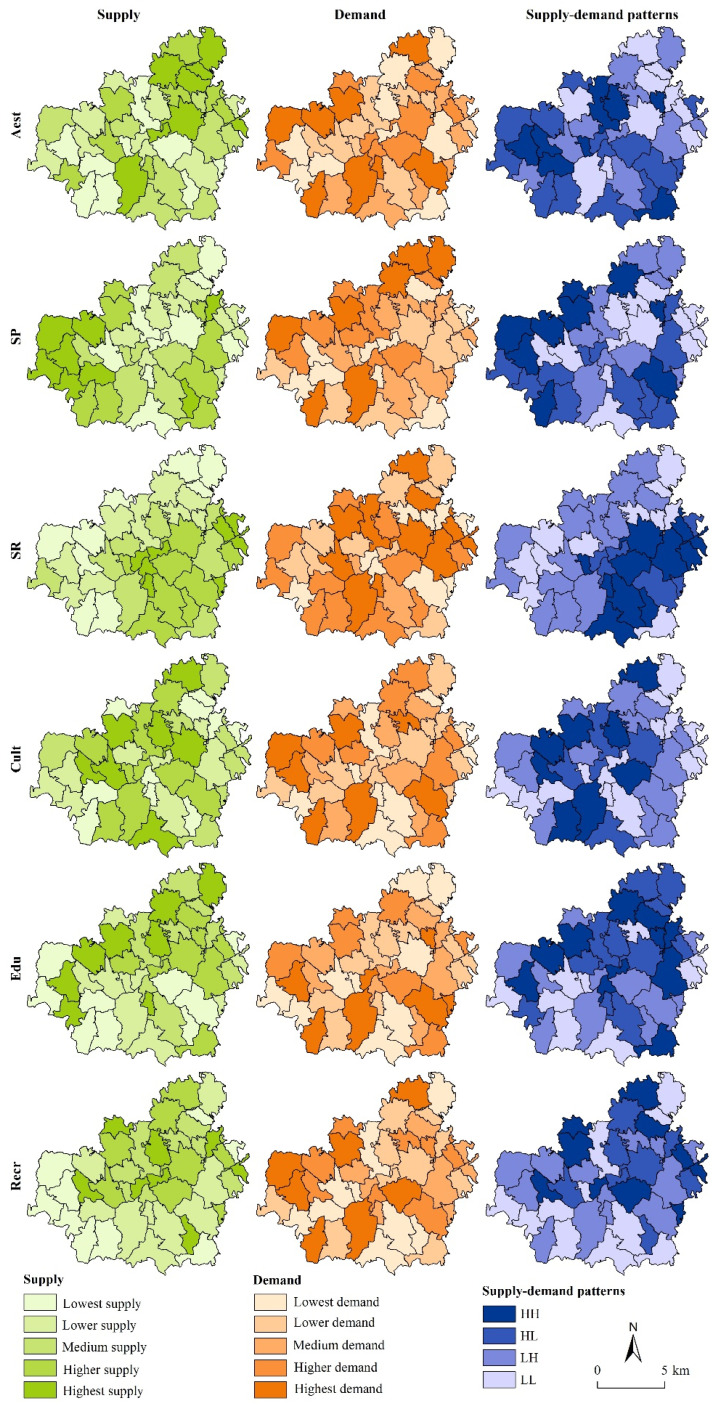
Spatial distribution of supply, demand and supply–demand patterns of cultural ecosystem services in Shigou Township. HH: high supply–high demand; HL: high supply–low demand; LH: low supply–high demand; LL: low supply–low demand.

The entropy weight method determines the weight of indicators based on the amount of information provided by the original values of each indicator, which can effectively solve the problem of information overlap between multiple indicators and make the evaluation result more objective [46]. In this study, the entropy weight method was used to determine the weight of each indicator of CES supply, and the weight calculation results are shown in Table 1. The specific calculation steps are as follows:

(1) $$(1)\;\mathrm{Standardization}:\;\mathrm{anticipated}\;\mathrm{impact}\;(+):\;X_{ij}^{\prime }=X_{ij}/\lambda_{j\max};$$

(2) $$\mathrm{anticipated}\;\mathrm{impact}\;(-):\;X_{ij}^{\prime }=\lambda_{jmin}/X_{ij}$$

(3) $$(2)\;\mathrm{Normalization}:\;Y_{ij}=X_{ij}^{\prime }/\overset{n}{\underset{i=1}{\sum }}X_{ij}^{\prime }$$

(4) $$(3)\;\mathrm{Indicator}\;\mathrm{information}\;\mathrm{entropy}:\;e_{j}=\left(-\frac{1}{\ln n}\right)\times \overset{n}{\underset{i=1}{\sum }}Y_{ij}\ln Y_{ij}$$

(5) $$(4)\;\mathrm{Indicator}\;\mathrm{difference}\;\mathrm{coefficient}:\;d_{j}=1-e_{j}$$

(6) $$(5)\;\mathrm{Indicator}\;\mathrm{weight}:\;w_{j}=d_{j}/\overset{m}{\underset{j=1}{\sum }}d_{j}$$

(7) $$(6)\;\mathrm{CES}\;\mathrm{supply}\;\mathrm{index}:\;S_{k}^{a}=\overset{m}{\underset{j=1}{\sum }}w_{j}Y_{ij}^{k}$$

where $X_{ij}$ is the original value of row i and column j; $\lambda_{jmax}$ and $\lambda_{jmin}$ are the maximum and minimum of the original value of j column, respectively; n is the number of sample administrative villages; m is the number of indicators of each CES; $S_{k}^{a}$ is the supply index of a type of CES in k administrative village; $Y_{ij}^{k}$ is the normalized value of row i and column j in k administrative village; and a refers to the six types of CESs investigated in the study. The calculated supply index was classified into five grades by the quantile method using ArcGIS 10.4 software (Environmental Systems Research Institute, Redlands, CA, USA), with reference to a study by Wang et al. [61], including the highest supply, higher supply, medium supply, lower supply and the lowest supply. Figure 2 shows the spatial distribution of the CES supply in Shigou Township.

#### 2.3.2. Quantifying the CES Demand

(1) CES demand scale

At present, scholars define CES demand from the perspectives of consumption, society and individual preference, and it is more appropriate to use keywords such as “expectation” and “preference” to understand the demand of non-material CES [14,62]. We referred to studies by MEA [1], Sherrouse et al. [16], Plieninger et al. [40], Angarita-Baéz et al. [10] and Dou et al. [63] to design a CES demand scale for this study. Combining the actual situation of Shigou Township, six types of CES were selected as our research objects, including Aest, SP, SR, Cult, Edu and Recr. The CES demand scale for Shigou Township is shown in Table 2.

(2) Calculating the CES demand

Based on the research of Ciftcioglu [15] and Wang et al. [64], the average value of respondents’ perceived importance of the six types of CES represents the CES demand in the study area. The calculation formula is as follows:

(8) $$D_{k}^{a}=\frac{1}{\beta }\overset{\beta }{\underset{j}{\sum }}Y_{k}^{\alpha }$$

where $D_{k}^{a}$ refers to the a type of CES demand index in k administrative village, $Y_{k}^{\alpha }$ refers to the perceived importance value of respondent α to the a type of CES in k administrative village and β refers to the number of respondents in k administrative village. The calculated demand index was classified into five grades by the quantile method using ArcGIS 10.4 software, with reference to a study by Wang et al. [61], including the highest demand, higher demand, medium demand, lower demand and the lowest demand. Figure 2 shows the spatial distribution of the CES demand in Shigou Township.

#### 2.3.3. Identifying Imbalance in Supply and Demand of CES

The commonly used method for calculating imbalance in the supply and demand of ESs is the ecological supply–demand ratio, which is applicable when the supply and demand units are consistent [34,38,65]. Because the units of supply and demand of CES are not consistent, it is unreasonable to directly calculate the supply–demand ratio to pursue an absolute surplus and deficit in quantity [53,66]. To explore the imbalance in the supply and demand of CES in Shigou Township, Z-score standardization was used to standardize the supply and demand indices to determine the balancing pattern of CES supply and demand [61,67]. The standardized supply index is plotted on the x-axis, and the standardized demand index is plotted on the y-axis, which generates four quadrants: high supply–high demand (HH), low supply–high supply (LH), low supply–low demand (LL) and high supply–low demand (HL). The calculation formulae are as follows:

(9) $$x=\frac{x_{i}-\overset{-}{x}}{s}$$

(10) $$\overset{-}{x}=\frac{1}{n}\overset{n}{\underset{i=1}{\sum }}x_{i}$$

(11) $$s=\sqrt{\frac{1}{n}\overset{n}{\underset{i=1}{\sum }}{\left(x-\overset{-}{x}\right)}^{2}}$$

where x is the standardized supply index or demand index, $x_{i}$ is the supply index or demand index of the *x*-th evaluation village, $\overset{-}{x}$ is the average of all villages, s is the standard deviation of all villages and n is the total number of villages. The four quadrant diagrams of the six types of CES supply and demand are in available in the Supplementary Material (Figures S1–S6)), and the spatial distributions of the CES supply–demand patterns in Shigou Township are shown in Figure 2 [61,67].

#### 2.3.4. Identifying Tradeoffs/Synergies on the CES Supply Side

The bivariate local Moran’s I in GeoDa software (The University of Chicago, Chicago, IL, USA) was used to perform a bivariate spatial autocorrelation analysis of the CES supply index. This method can not only numerically reflect the tradeoffs/synergies between various CESs but also present the spatial agglomeration of tradeoffs/synergies between various CESs. In the analysis results, HH and LL clustering indicate a synergistic relationship, and HL and LH clustering indicate a trade off relationship [68].

#### 2.3.5. Identifying CES Demand Differences between Stakeholder Groups

According to the actual situation of Shigou Township and respondents’ occupations, all respondents were classified into five groups of stakeholders: farmers, migrant workers, multiple occupations, elderly people and government staff. Farmers mainly rely on plantations as a source of income. Migrant workers are respondents who leave the village to work in other places. Multiple occupations refers to simultaneous engagement in two or more means of livelihood, such as planting and breeding or planting and working. Elderly people are defined as those more than 60 years old who receive income from national pensions. Government staff refers to local leaders, such as village chiefs, accountants and directors. Chi-square tests of independence were used to test the differences in demand for the six types of CES among stakeholders [69].

## 3. Results

### 3.1. Spatial Imbalance in the Supply and Demand of CES

#### 3.1.1. CES Supply

The CES supply in Shigou Township presents evident spatial differences (Figure 2). Among them, Aest presents a spatial pattern of high supply in the northeast and low supply in the southwest. High-supply villages of SP are distributed in the west, whereas low-supply villages are distributed in the east. The SR supply is high in the central and eastern villages but low in the northern and western villages. Cult presents a spatial pattern of high supply in the central villages and a low supply in both the east and the west; the proportion of the highest-supply villages is smallest, at 16.67%. Edu supply is high in the north and low in the south. Recr supply is high in the northeast and low in the west and south. In terms of Aest, SP, SR, Edu and Recr, the villages with lower and the lowest supply account for the largest proportion, each accounting for 21.43%.

#### 3.1.2. CES Demand

The basic characteristics of all respondents in terms of CES demand are shown in Table S2. The sample includes 63 women (16.5%) and 318 men (83.5%).In terms of the age of respondents, the age group 61–70 years accounts for the largest proportion (33.6%), whereas the proportion of other age groups (19–40, 41–50, 51–60, >70 years old) account for 6.3%, 11.8%, 32.3% and 16.0% of the total sample, respectively. In terms of educational background, respondents with middle school education account for the largest proportion (39.9%), followed by primary school, college education and uneducated, accounting for 33.3%, 25.5% and 1.3%, respectively.

Spatial differences in the CES demand in Shigou Township are also evident (Figure 2). Among them, Aest presents a spatial pattern of high demand in the periphery and low demand in the central region; the villages with lower and the lowest demand account for the largest proportion, each accounting for 21.43%. The SP demand is high in the marginal villages of the northeast, northwest and southwest and low in the central and southeast villages; the proportion of the highest-demand villages is the smallest, at 16.67%. The lowest-demand villages of SR are scattered along the eastern edge, the central area and the western edge of the study area. Furthermore, the proportion of the highest- and higher-demand villages is the largest, each accounting for 26.19%. The spatial distribution pattern of Cult demand is similar to that of Aest demand, and the proportion of lower-demand villages is the largest, at 28.57%. Edu and Recr present a staggered spatial distribution of high-demand villages, moderate-demand villages and low-demand villages. The proportions of lower-demand villages of Edu and Recr are the largest, at 26.19% and 23.81, respectively.

#### 3.1.3. The Supply–Demand Patterns of CES

The balanced supply–demand patterns of CES in Shigou Township are shown in Figure 2. For Aest, the HH and HL, patterns are mainly located in the west and southeast, each accounting for 23.81%; the LH and LL patterns account for 33.33% and 19.05%, respectively and are mainly located in the northeast. For SP, the HH and HL patterns account for 21.43% and 26.19%, respectively, and include villages on the periphery of Shigou Township. The LH and LL patterns account for 21.43% and 30.95%, respectively, and are mainly located in the central and eastern regions on the township. For SR, the HH and HL patterns account for 26.19% and 14.29%, respectively, and are located in the southeast. The LH and LL patterns account for 33.33% and 26.19%, respectively, and occupy more than half of Shigou Township in a northeast–southwest trend. For Cult, the HH and HL patterns account for 19.05% and 11.90%, respectively, and are located in the central region of the township; the LH and LL patterns account for 30.95% and 38.10%, respectively, and are located in the east and west. For Edu, the HH and HL patterns are located in the northeast, and their proportions are the same (26.19%); the LH and LL patterns account for 26.19% and 21.43%, respectively, and are located in the south. For Recr, the HH and HL patterns account for 23.81% and 19.05%, respectively, and are located in the northeast; the LH and LL patterns account for 30.95% and 26.19%, respectively, and are located in the south and the northeast corner of the township.

### 3.2. Tradeoffs/Synergies on the CES Supply Side

**Figure 3:**
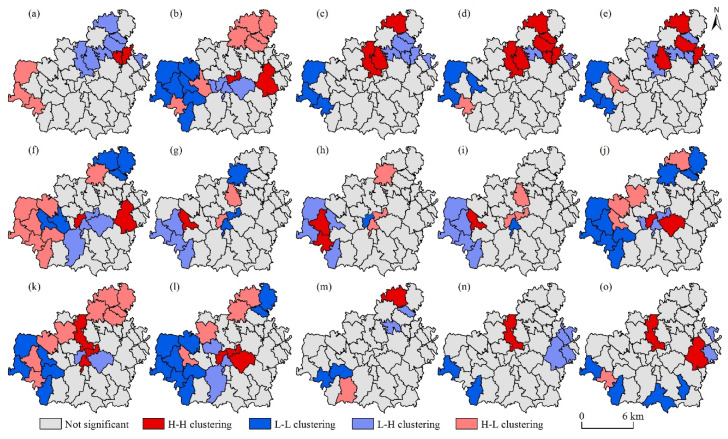
Spatial agglomeration of tradeoffs/synergies between six types of cultural ecosystem services on the supply side. (**a**): Aest-SP; (**b**): Aest-SR; (**c**): Aest-Cult; (**d**): Aest-Edu; (**e**): Aest-Recr; (**f**): SP-SR; (**g**): SP-Cult; (**h**): SP-Edu; (**i**): SP-Recr; (**j**): SR-Cult; (**k**): SP-Edu; (**l**): SP-Recr; (**m**): Cult-Edu; (**n**): Cult-Recr; (**o**): Recr-Edu. Aest: aesthetic; SP: sense of place; SR: social relations; Cult: cultural heritage; Edu: education; Recr: recreation. H-H clustering: high–high clustering; L-L clustering: low–low clustering; L-H clustering: low–high clustering; H-L clustering: high–low clustering.

Bivariate spatial autocorrelation analysis was performed on the six types of CES supply, and the local autocorrelation Moran’s I (Table 3) and the spatial clustering maps of tradeoffs/synergies on the CES supply side were obtained (Figure 3). Table 3 shows that numerically, the tradeoffs and synergies among the six types of CES supply coexist. The tradeoff relationships include Aest-SP, SR-SP, Cult-SP, Edu-SP, Recr-SP, SR-Cult and SR-Edu. The synergistic relationships include Aest-SR, Aest-Cult, Aest-Edu, Aest-Recr, SR-Recr, Cult-Recr, Cult-Edu and Edu-Recr.

According to the spatial distribution (Figure 3), the southwest, northeast, and central parts of Shigou Township are the key regions of tradeoffs/synergies of the CES supply. First, in the southwest of Shigou Township, there is a tradeoff relationship of HH clustering between the SP supply and the other five types of CES supply, whereas there is a synergistic relationship of LL clustering among Aest, SR, Cult, Edu and Recr. Secondly, in the northeast, the CES supply presents as a synergistic relationship of HH clustering for Aest-SP and Aest-Recr; a tradeoff relationship of HL clustering for Aest-SR, Aest-Cult and Aest-Recr; a tradeoff relationship of LH clustering for Aest-SP, SR-Edu and SR-Recr; and a synergistic relationship of LL clustering for SP-SR, SR-Cult and SR-Recr. Thirdly, in the central region, the CES supply presents as a synergistic relationship of HH clustering for SR-Recr; a tradeoff relationship of HL clustering for SR-Recr and SR-Edu; a tradeoff relationship of LH clustering for Aest-SR, SP-SR, SR-Edu and SR-Recr; and a synergistic relationship of LL clustering for SP-Cult.

### 3.3. CES Demand Difference among Stakeholder Groups

The chi-square test results show that there are significant differences in the three types of CES demand among stakeholder groups, namely social relations (χ^2^ = 23.735, *p* = 0.095), cultural heritage (χ^2^ = 30.248, *p* = 0.017) and education (χ^2^ = 24.882, *p* = 0.072) (Table 4). Government staff have a higher demand for social relations and cultural heritage than other groups. Specifically, 44.8% and 31.0% of government staff consider social relations and cultural heritage, respectively, to be “very highly important”. Individuals with multiple occupations have a higher demand for education than other groups, and 20.5% of respondents with multiple occupations consider education to be “very highly important”. In addition, the chi-square test results show that the differences in Aest, SP, and Recr between the five stakeholder groups do not pass the significance test (Table 4).

## 4. Discussion

### 4.1. Quantitative Method of CES Supply and Demand

In the studies of Church et al. [6] and Tratalos et al. [49], the method of biophysical indicators was proven to be a useful tool for quantifying CES supply. An indicator can be defined as a measurement based on verifiable data that transmits more information than the indicator itself [48]. However, CES research on biophysical indicator methods on a small scale is still lacking [70]. Rural ecosystem management should be implemented into the basic unit of administrative management: villages. Therefore, conducting a CES supply-and-demand assessment on a small scale at the village level is of considerable significance for understanding the interaction between ecosystems and human well-being [71]. In this study, we demonstrated the method of quantifying and spatially displaying the CES supply and demand on a village scale. CES does not represent a purely ecological phenomenon but is the result of a complex and dynamic relationship between ecosystems and humans over a long time span [40]. The CES of a place has specific connections with specific ecosystem characteristics, the land-use patterns determine the intensity of the CES supplied by the ecosystem and a CES value can be assigned to specific land uses related to human activities [27]. The physical characteristics of land use indicate only the supply potential of CES, whereas the actual supply of CES also depends on its availability to the local population [49]. Therefore, land use and accessibility are important components of a CES supply assessment [35]. Furthermore, the quality of environmental space is spatially heterogeneous; for example, the contribution of landscape heterogeneity to CES varies. Therefore, quality should also be included in the indicator system of CES assessment [49]. Based on the actual situation of Shigou Township, in this paper, we constructed an indicator system of CES supply based on the three aspects of supply potential, accessibility and quality. Typical land uses in Shigou Township include cropland, woodland, grassland, water bodies, residential land, scenic land and special cultural landscapes, including cultural activity centers and temples, which are regarded as potential supply indicators [6]. The distances from residential locations to various land uses and cultural landscapes are regarded as accessibility indicators [72], and landscape heterogeneity is regarded as an indicator of quality [40].

Some studies have noted that the boundaries are not clear, and there may be overlaps between CES categories [12,56], which could account for why current CES studies focus on specific subcategories rather than on an overall, comprehensive CES scope [73]. However, all services are equally important and must be evaluated in a CES assessment [12]. Avoiding double counting and assigning different land uses to specific CES types is an operational approach [48]. For example, when the NDVI is regarded as a proxy indicator of aesthetic supply, as in this study, it is no longer included as an indicator of recreational supply. Therefore, it is necessary to acknowledge that, given the actual situation of CES evaluation, CES measurement is inevitably inaccurate [49]. In any case, we have proposed a series of indicators for CES supply. These indicators can quantify and spatialize various types of CESs on different scales and represent a useful contribution to quantitative research on CES.

CESs are closely linked to human experiences and feelings [7], and understanding how people perceive CES is the basis of ecosystem management [27]. Questionnaires are the most commonly used non-monetary evaluation method in CES demand assessment [12] and are superior for small-scale data collection. The combination of the biophysical indicator method and questionnaires makes small-scale CES supply-and-demand research possible from the perspective for a comprehensive understanding of the relationship between humans and ecosystems.

### 4.2. Spatial Distribution Characteristics of CES Supply and Demand

The supply and demand of CES may vary geographically, and this heterogeneity requires mapping [35]. Consistent with the findings of Peña et al. [74] and Depietri et al. [28], in our study, we also identified the importance of infrastructure and accessibility to the supply of Recr. In addition, in Shigou Township, superior infrastructure and accessibility also have a positive impact on the supply of SR. Shigou Township is a flat valley in the central region, with high accessibility of settlements to various landscapes, limited separation of settlements and close distances from villages to towns and counties; the villages located in the center are convenient for internal and external contacts and therefore have a high supply of SR and Recr. This characteristic further proves that SR and Recr have high accessibility requirements. The influence of topography and land use on CES supply has been confirmed by numerous studies [23,75,76]. In our study, we found that in the northeast of Shigou Township, the terrain is relatively flat, and the quality of cultivated land is high. Farmers only need to maintain high-quality farmland, such as dam land, to ensure grain yield, whereas slopes and mountain land are converted to forest and orchards [31], with high vegetation coverage, which provide natural resources for the supply of Aest, SP and Recr. Therefore, Aest, SP and Recr in northeastern Shigou Township exhibit synergistic relationships of HH clustering. The terrain in the southwest of Shigou Township is dominated by mountainous hills, with poor land resource endowment. More cultivated land resources are formed by extensive expansion by farmers, which is necessary to sustain their livelihoods [46]. These human activities have negative ecological effects, such as vegetation destruction and soil erosion, coupled with topographical restrictions [45]. The layout of residential locations is chaotic, and accessibility is poor, which is not conducive to the supply of CESs, such as Aest, SR and Recr. This also explains why in the southwest of Shigou Township, synergistic relationships were found for LL clustering between Aest, SP, SR, Edu and Recr.

A study by Scully-Engelmeyer et al. [77] revealed that coastal residents are more likely to perceive the CES provided by coastal resources than residents in other areas, indicating that regional differences are reflected in CES research, even within a small range. Our research also revealed the impact of location conditions on CES demand and regional differences in CES demand. The spatial heterogeneity of the CES demand in Shigou Township is related to villages’ industrial development and location conditions. For example, residents of Liujiagua, with a developed tourism industry, have the strongest perceived importance of Aest. The east of Shigou Township is adjacent to the county, and the values of residents in the east are impacted by urbanization. Residents’ demand for SP in the east is lower than that of residents in the west, who are far from the county. In fact, all physical, social and economic factors affect residents’ demand for CES [30].

Avoiding conflict between stakeholders is an important part of ecosystem management decisions [69]. A study by García-Nieto et al. [78] found that policy-relevant stakeholder groups, such as environmentalists or government members, had different perceptions of CES compared to stakeholder groups with direct land linkages, such as farmers or hunters. Our research revealed a similar trend, i.e., that village cadres’ perception of SR and Cult is higher than that of farmers, migrant workers, those with multiple occupations and elderly people. Therefore, consultation with different stakeholder groups is required for management decisions in order to reduce tradeoffs for specific services.

### 4.3. Limitations

In this study, we evaluated CES supply and demand by coupling biophysical indicators and questionnaire methods at the village scale, which proved an effective method for research on supply and demand matching of CES at the microscale. The data used in this study can also be obtained in other regions, with the potential to expand this approach in other regions. However, this study also has some limitations. First, we evaluated the spatial pattern of CES supply and demand without considering their temporal changes. Research on the temporal change in CES supply and demand can enrich our understanding of CES supply and demand matching and provide suggestions for alleviating the mismatch [79]. Secondly, our research on CES supply and demand is only limited to local areas, without consideration of any type of CES flow. CES flow provides possible time and space mismatch information between supply areas and benefit areas [66]. Thirdly, in the assessment of CES demand, due to multiple factors, such as the time and manpower required for the questionnaire survey, the uneven distribution of respondents in each village or the inconsistent understanding of the same concept of CES by respondents in different places, there may be some bias in the data [10]. In future research, the questionnaire should span a longer time in order to collect more samples to prevent subjective bias.

### 4.4. Policy Implications

Our study revealed that land-use differences caused by the complex topography of Shigou Township have become potential factors for the spatial heterogeneity of the CES supply and demand, as well as and tradeoffs/synergies. The western terrain of Shigou Township is dominated by mountains and hills, which are disturbed by human activities during agricultural production [45]. This method of extensively expanding cultivated land to strengthen people’s local attachment is unsustainable. Comprehensive land management should be implemented in this region. For example, grain crops should be planted in relatively flat areas, and the beautification of farmland should be considered at the same time. In addition, the layout of residential locations should be reasonably planned in flat areas. The conversion of farmland to forest should be carried out on slopes. The transformation of wide terraces should be carried out in mountainous areas, and economic forests (such as apple trees and apricot trees suitable for the local climate) should be planted in this area. Roads should also be built around mountains to facilitate the transportation of agricultural products and improve the supply of aesthetic and recreational services [80]. The central and eastern regions of Shigou Township have the advantages of being adjacent to the county and flat terrain, and various CESs have a high supply. However, we noticed that people in this region have a lower demand for sense of place, which may be because urbanization is threatening many planting systems and traditional cultures with high ecological, historical and cultural values [9]. Therefore, this region should consider CES supply and combine local characteristics of culture to build ecological and cultural bases based on aesthetic sightseeing, ecotourism and traditional farming culture to avoid a lack of sense of place associated with the wave of urbanization.

We included stakeholders in the CES evaluation and found that the differences in CES demand among stakeholder groups in Shigou Township were related to their living conditions and work experience. Government staff are direct participants in policy making and implementation and play an important role in co-ordinating the internal and external interest conflicts of villages, so they can realize the importance of social relations and cultural heritage [69]. However, farmers and migrant workers are busy with their livelihoods and pay less attention to social relations and cultural heritage. Therefore, policy makers should take advantage of local resources to increase employment of farmers and migrant workers by planting characteristic agricultural products, extending the agricultural product industrial chain, developing rural tourism and publicizing the importance of ecological and cultural values on the basis of solving poverty problems [80]. Most of those with multiple occupations in Shigou Township use a combination of planting, economic forest and breeding as their source of income. They have more direct contact with the ecosystem and can realize the importance of reasonable land use and ecological protection; that is, they can realize the importance of educational services. Ecosystem knowledge can shape people’s attitudes and behavioral intentions [76]. Policy makers should involve such stakeholders in ecosystem management by engaging them in specific forest protection work or training them as propagandists of ecological protection, ultimately improving the awareness of ecological protection in the whole region.

## 5. Conclusions

The gap in the evaluation and mapping of the CES supply and demand in ES research has become the most important restriction hindering the role of ESs in ecosystem management decisions. Using Shigou Township, Mizhi County, China, as an example, in this study, we evaluated the CES supply and demand at the village scale. This study proved the superiority of the combination of the biophysical indicator method and questionnaire method for the assessment of CES supply and demand on a small scale. The results show that CES supply and demand presented evident spatial differences, and the land-use differences caused by the complex topography of Shigou Township are potential factors impacting the spatial heterogeneity of the CES supply and demand, as well as tradeoffs/synergies. On the CES supply side, the southwest, northeast and central areas of Shigou Township are the key regions of tradeoffs/synergies of CES supply. On the demand side, there are significant differences in CES demand for social relations, cultural heritage and education among stakeholder groups. Based on the research results, we suggest that policy makers should carry out comprehensive land management in the west of Shigou Township, considering regional advantages and building ecological and cultural bases based on aesthetic sightseeing, ecotourism and traditional farming culture in the central and eastern regions of the township. In addition, we suggest that policy makers should consider the demands of different stakeholders and encourage them to participate in ecosystem management.

## Figures and Tables

**Table 1:** Indicator system of cultural ecosystem services in Shigou Township.

CES	Indicator	Data Processing (Units)	Indicator Type	Anticipated Impact	Weight
Aesthetic	NDVI	ArcGIS 10.4 zonal statistics	Quality	+	0.500
	SHDI	Fragstats 4.2 (−)	Quality	+	0.500
Sense of place	Per capita cropland area	Ratio of cropland area to total population (m^2^/person)	Supply	+	0.499
	Per household cave dwelling area	Ratio of cave dwelling area to total number of households (m^2^/household)	Supply	+	0.501
Social relations	Residential separation	Fragstats 4.2 (−)	Quality	−	0.345
	Distance to the nearest township	ArcGIS 10.4 proximity analysis (m)	Accessibility	−	0.312
	Distance to county	ArcGIS 10.4 point distance (m)	Accessibility	−	0.343
Cultural heritage	Cultural activity center area	Field interviews (m^2^)	Supply	+	0.520
	Temple area	Field interviews (m^2^)	Supply	+	0.480
Education	Percentage of “three types of land” area	Ratio of “three types of land” area to total cropland area (%)	Supply	+	0.500
	Percentage of “Grain for Green” area	Ratio of “Grain for Green” area to total cropland area (%)	Supply	+	0.500
Recreation	Percentage of scenic spots area	ArcGIS 10.4 statistical analysis (%)	Supply	+	0.131
	Shortest distance to woodlands	ArcGIS 10.4 proximity analysis (m)	Accessibility	−	0.218
	Shortest distance to grasslands	ArcGIS 10.4 proximity analysis (m)	Accessibility	−	0.218
	Shortest distance to water bodies	ArcGIS 10.4 proximity analysis (m)	Accessibility	−	0.216
	Average distance to cultural activity center	ArcGIS 10.4 point distance (m)	Accessibility	−	0.217

Anticipated Impact: “+” indicates that the indicator is positively correlated with the CES supply; “−“ indicates that the indicator is negatively correlated with the CES supply.

**Table 2:** Cultural ecosystem services demand scale in Shigou Township and the references sources.

CES		Reference Source
Aesthetic	The beauty I can get from the landscapes in my village, such as beautiful scenery, rich colors, etc.	[16]
Sense of place	My village is special to me; it makes me feel safe, comfortable and attached.	[63]
Social relations	My village has had an important impact on a variety of specific social relations.	[1,40]
Cultural heritage	I think landscapes with historical and cultural value are important to the inheritance of traditional culture.	[10]
Education	The land use and ecological protection measures in my village provide me with a source of informal education.	[10]
Recreation	I like leisure activities, such as chatting, walking, dog walking, playing with children, exercising, etc.	[63]

**Table 3:** Bivariate local Moran’s I among six cultural ecosystem service supplies in Shigou Township.

CES	Aesthetic	Sense of Place	Social Relations	Cultural Heritage	Education	**Recreation**
Aesthetic	1.000					
Sense of place	−0.270	1.000				
Social relations	0.101	−0.273	1.000			
Cultural heritage	0.032	−0.025	−0.052	1.000		
Educational	0.178	−0.200	−0.051	0.056	1.000	
Recreation	0.149	−0.203	0.126	0.067	0.129	1.000

**Table 4:** Cultural ecosystem service demand among five different stakeholder groups in Shigou Township.

CES	χ^2^ (*p*)	Farmer (*n* = 161) (%)	Migrant Worker (*n* = 33) (%)	Multiple Occupations (*n* = 73) (%)	Elderly People (*n* = 85) (%)	Government Staff (*n* = 29) (%)	Total (*n* = 381) (%)
Aesthetic	19.350 (0.251)	39.1	33.3	50.7	37.6	34.5	40.2
Sense of place	21.265 (0.169)	43.5	33.3	58.9	56.5	48.3	48.8
Social relations	23.735 (0.095)	28.0	30.3	41.1	34.1	44.8	33.3
Cultural heritage	30.248 (0.017)	16.1	6.1	28.8	18.8	31.0	19.4
Education	24.882 (0.072)	19.9	15.2	20.5	16.5	3.4	17.6
Recreation	10.639 (0.831)	16.1	9.1	16.4	14.1	17.2	15.2

## Data Availability

Land-use data can be acquired from the Chinese Academy of Sciences Resource Environmental Data Center. Normalized difference vegetation index (NDVI) data can be derived from Landsat 8 remote-sensing images, which are openly available. Questionnaires and interview data are contained within the Supplementary Materials.

## Supplementary Materials

The following supporting information can be downloaded at: https://www.mdpi.com/article/10.3390/ijerph19116661/s1, Table S1: Serial number (SN) of villages corresponding to Figure 1 and respondent number (N) in 42 villages of Shigou Township; Questionnaire S1: Questionnaire form developed for “assessment of the demand of cultural ecosystem services”; Questionnaire S2: Main interview questions used to obtain CES supply data; Table S2: Characteristics of respondents with respect to the demand for cultural ecosystem services. Figure S1: Quadrant diagram distribution of supply–demand patterns of Aest; Figure S2: Quadrant diagram distribution of supply–demand patterns of SP; Figure S3: Quadrant diagram distribution of supply–demand patterns of SR; Figure S4: Quadrant diagram distribution of supply–demand patterns of Cult; Figure S5: Quadrant diagram distribution of supply–demand patterns of Edu; Figure S6: Quadrant diagram distribution of supply–demand patterns of Recr.
