# Supplementary material for: Identifying the Spatial Imbalance in the Supply and Demand of Cultural Ecosystem Services
Source: Int J Environ Res Public Health. 2022 May 30;19(11):6661. doi: 10.3390/ijerph19116661 (PMC9180195; doi:10.3390/ijerph19116661)
Supplement: Supplementary file 1 [file ijerph-19-06661-s001.zip › ijerph-1712488-supplementary.pdf]

Supporting Materials A: The serial number (SN) of villages and respondents number (N) in 42 villages of Shigou Township.

Table S1: The serial number (SN) of villages corresponding to figure 1 and respondents number (N) in 42 villages of Shigou Township.

SN	Village	N	SN	Village	N	SN	Village	N
1	Aihaowan	10	15	Laoyan	8	29	Songshan	10
2	Cuigelao	7	16	Lijiagua	6	30	Womaping	13
3	Dangjiata	9	17	Lishengyan	11	31	Xiaiqu	10
4	Dangping	10	18	Liujiagou	10	32	Xichangjiaqu	10
5	Dongshanliang	6	19	Liujiagua	10	33	Xichangxingzhuang	13
6	Dujiashigou	10	20	Loujiagua	7	34	Xidujiagou	8
7	Duxinzhuang	10	21	Miaoshan	5	35	Xigaoqu	12
8	Fengjian	7	22	Miaoyan	12	36	Ximajiagou	11
9	Gaojiagua	10	23	Pancaogou	5	37	Yanglugou	11
10	Gaoxingzhuang	11	24	Pangfu	9	38	Yangpan	9
11	Guandaoshan	11	25	Renjiaping	7	39	Yanjiapan	7
12	Haojiaxingzhuang	10	26	Shanjiagou	12	40	Yuanyangshan	11
13	Heishanze	5	27	Shushan	8	41	Zhangjiapan	9
14	Heiyanqing	9	28	Silanggou	7	42	Zhuxingzhuang	5

Questionnaire S1: The questionnaire form developed for "assessment of the demand of cultural ecosystem services".

Introduction

Excuse me, I am a graduate student at College of Urban and Environmental Science, Northwest University. I would like to invite you to take part in our research study, which concerns your perceived importance (preference) of cultural ecosystem services (CES) provided by the local ecosystem. In short, the CES are defined as "ecosystems" contributions to the non-material benefits that arise from human-ecosystem relationships. For example, you value some sites because you enjoy the scenery, sights, colors, sounds, smells, etc. Then, I will give you some statement, could you please tell how much you agree with me. It should last about 30-40mins. In addition, our questionnaire is anonymous and your personally identifiable information will be handled as confidentially as possible.

Section I: Profile of the respondents

Name of village: ________________ Date: _______________

Age: _________ Gender: ①Female ②Male

Family population: __________ Annual income: _________RMB

Education level: ①Illiteracy; ②Primary school; ③Junior high school; ④Senior high; ⑤University

Family income sources: ①Farming; ②Feeding livestock; ③Planting economic forests; ④Wage income; ⑤Other

Section II: Valuation of CES demand using a 5-point Likert scale (1: very low importance; 2:

low importance; 3: medium importance; 4: high importance; 5: very high importance).

CESs	Degree of perceived importance of CES				
Aesthetic	1	2	3	4	5
Educational	1	2	3	4	5
Sense of place	1	2	3	4	5
Social relations	1	2	3	4	5
Cultural heritage	1	2	3	4	5
Recreation	1	2	3	4	5

Questionnaire S2: Main interviews questions for obtaining some CES supply data.

The population of interviews are government staff of the village, such as village chiefs, accountants, and directors.

1. How many people are there in your village?
2. How many households are there in your village?
3. How much is the area of cave dwellings in your village?
4. How much is the area of temple in your village?
5. How much is the area of cultural activity center in your village?
6. How much is the area of "three types of land" (terraced, dam and irrigated land) in your village?
7. How much is the area of "Grain for Green" in your village?

Supporting Materials B:

Table S2: Characteristics of respondents about the demand of cultural ecosystem services.

Item	Group	Number	Proportion (%)
Gender	Male	318	83.5%
	Female	63	16.5%
Age	19-40	24	6.3%
	41-50	45	11.8%
	51-60	123	32.3%
	61-70	128	33.6%
	>70	61	16.0%
Education	Uneducated	97	25.5%
	Primary School	127	33.3%
	Middle School	152	39.9%
	≥College	5	1.3%

Supporting Materials C: Quadrant diagram distribution of supply-demand patterns of cultural ecosystem services in Shigou Township.

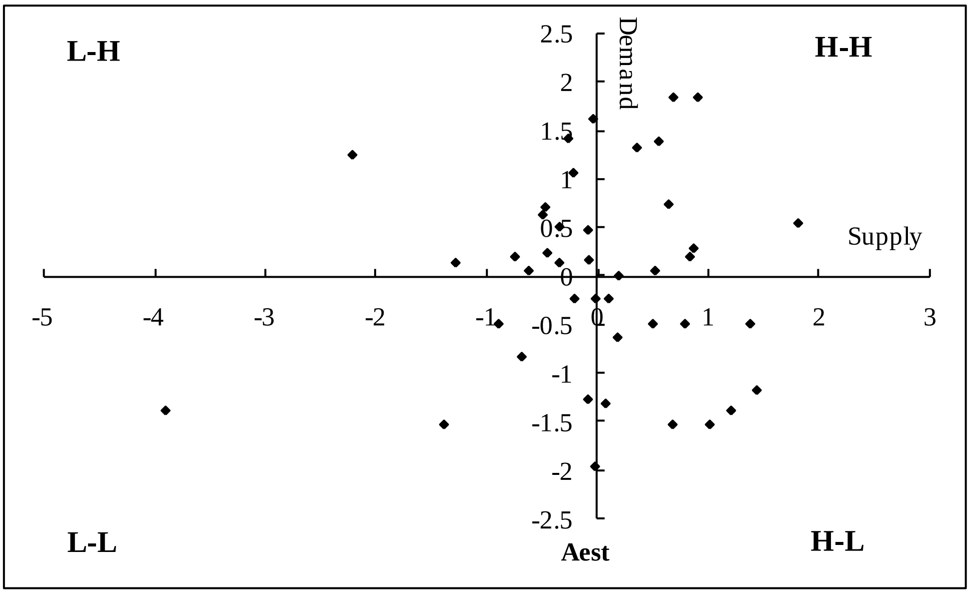

Figure S1. Quadrant diagram distribution of supply-demand patterns of Aest

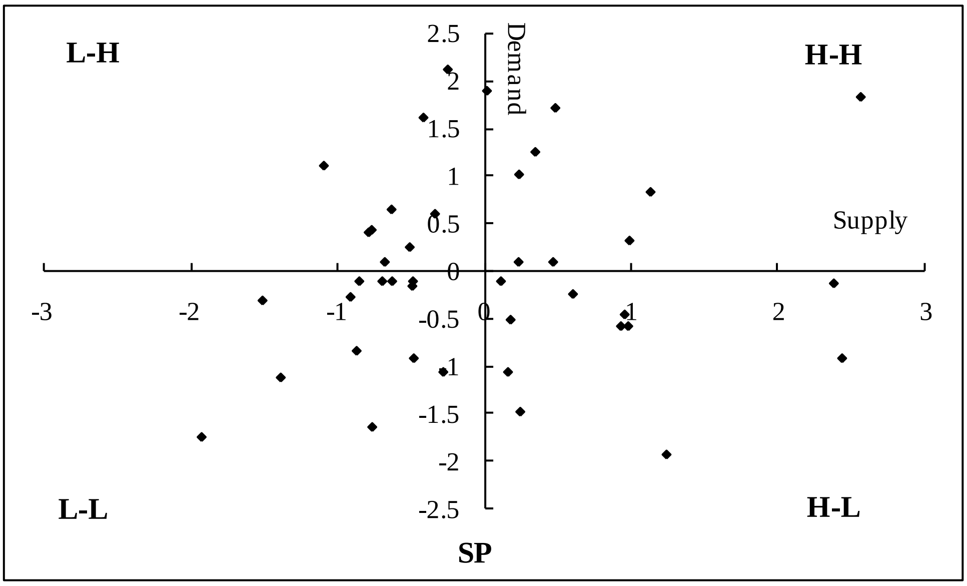

Figure S2. Quadrant diagram distribution of supply-demand patterns of SP

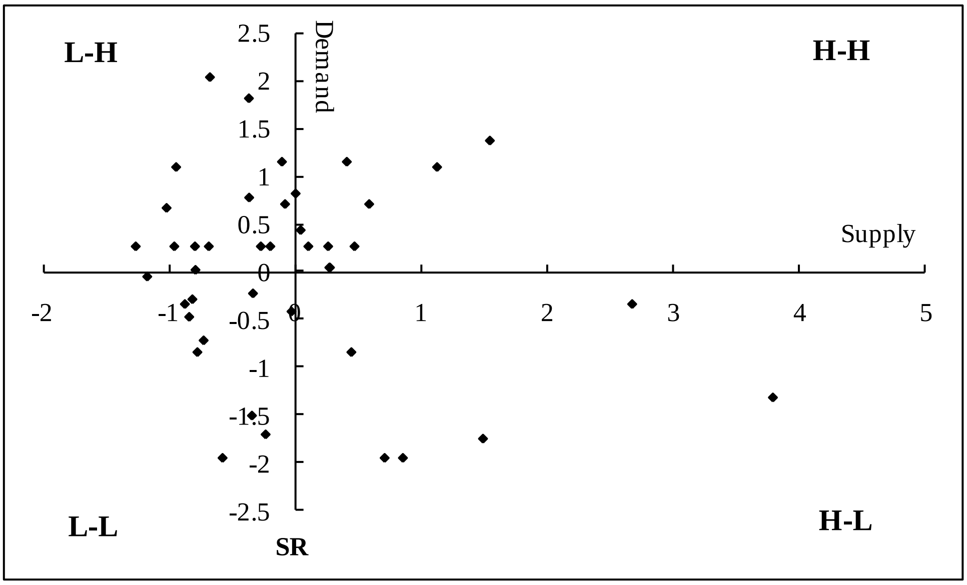

Figure S3. Quadrant diagram distribution of supply-demand patterns of SR

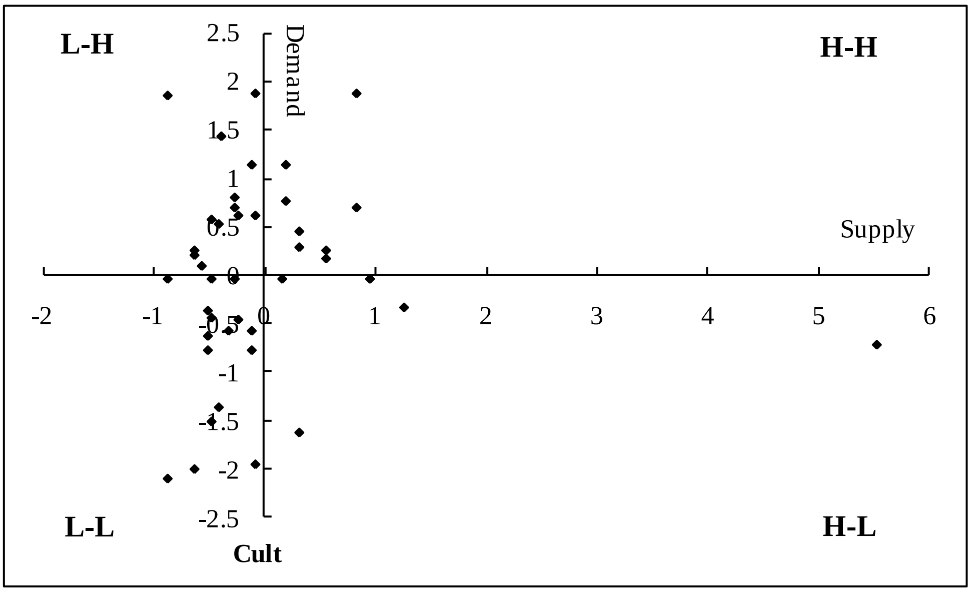

Figure S4. Quadrant diagram distribution of supply-demand patterns of Cult

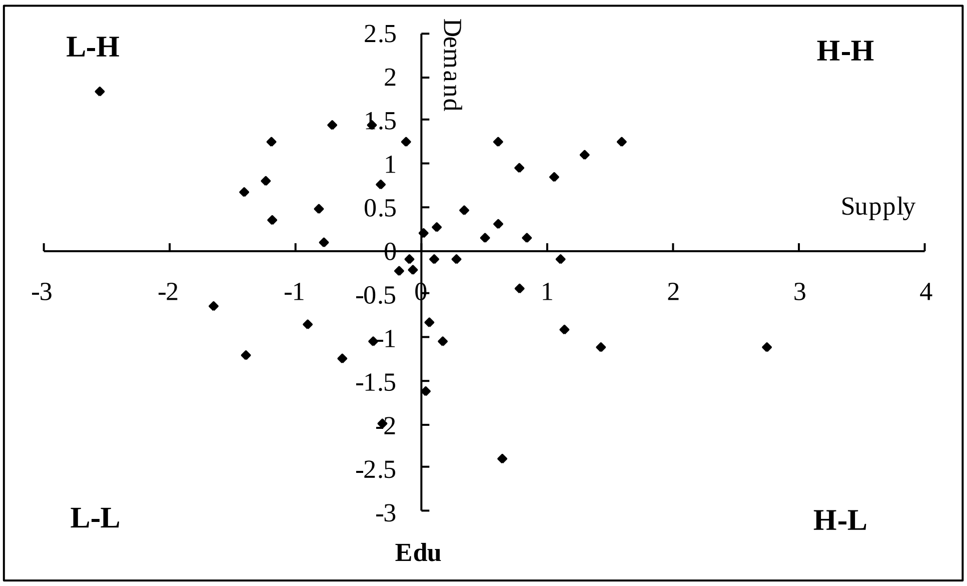

Figure S5. Quadrant diagram distribution of supply-demand patterns of Edu

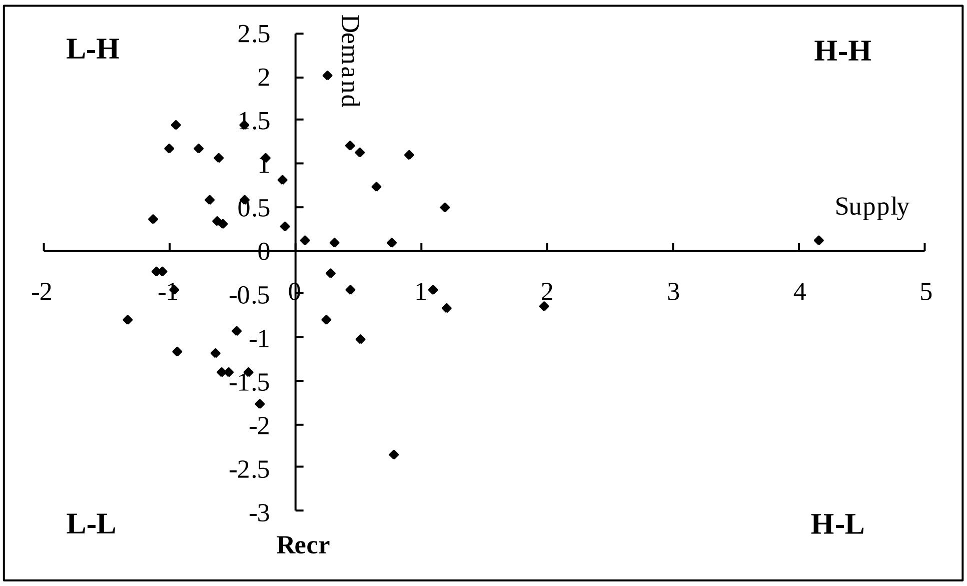

Figure S6. Quadrant diagram distribution of supply-demand patterns of Recr
